# CUX1 Facilitates the Development of Oncogenic Properties *Via* Activating Wnt/β-Catenin Signaling Pathway in Glioma

**DOI:** 10.3389/fmolb.2021.705008

**Published:** 2021-08-06

**Authors:** Fan Feng, Zongqing Zhao, Yunfei Zhou, Yanhao Cheng, Xiujie Wu, Xueyuan Heng

**Affiliations:** ^1^ Institute of Clinical Medicine College, Guangzhou University of Chinese Medicine, Guangzhou, China; ^2^ Institute of Brain Science and Brain-Like Intelligence, Linyi People’s Hospital, Linyi, China; ^3^ Department of Neurosurgery, Linyi People’s Hospital, Linyi, China

**Keywords:** CUX1, glioma, proliferation, cell cycle, wnt/β-catenin signaling, bioinformatics analysis

## Abstract

**Background:** Homeobox cut like 1 (CUX1), which often presents aberrated expression in many cancer cells, exerts a crucial role in tumorigenesis. Evidence describing CUX1 in gliomagenesis is scarce, and the effects of CUX1 on the Wnt/β-catenin pathway have not been reported. Our study aimed to explore the biological functions and molecular mechanisms involved in CUX1 activity in glioma.

**Methods:** Datasets for bioinformatics analysis were obtained from the GEO, TCGA, CGGA, GTEX and CCLE databases. qRT-PCR, western blotting (WB), and immunohistochemistry (IHC) assays were used to investigate the expression patterns of CUX1 among glioma and brain tissues. CUX1 knockdown and overexpression vectors were transfected into glioma cell lines, the CCK-8, clone formation assay, wound healing, Transwell assay, and flow cytometry were performed to detect changes in cell viability, invasiveness, and the cell cycle. WB and immunofluorescence (IF) assays were used to explore changes in cell cycle-related and Wnt/β-catenin signaling protein levels.

**Results:** Overexpression of CUX1 was identified in glioma tissues, and especially in glioblastoma (GBM), when compared to normal controls and correlated with poor prognosis. In comparison with untreated cells, TJ905 glioma cells overexpressing CUX1 showed higher proliferation and invasion abilities and S phase cell-cycle arrest, while the knockdown of CUX1 suppressed cell invasive ability and induced G1 phase arrest. Active Wnt/β-catenin signaling was enriched and clustered in a CUX1-associated GSEA/GSVA analysis. IF and WB assays indicated that CUX1 regulated the distribution of Axin2/β-catenin in glioma cells and regulated the expression of proteins downstream of the Wnt/β-catenin signaling pathway, suggesting that CUX1 served as an upstream positive regulator of the Wnt/β-catenin pathway. Finally, the knockdown of Axin2 or β-catenin could reverse the tumor-promoting effects caused by CUX1 overexpression, suggesting that CUX1 induced gliomagenesis and malignant phenotype by activating the Wnt/β-catenin signaling pathway.

**Conclusion:** Our data suggested that the transcription factor CUX1 could be a novel therapeutic target for glioma with gene therapy.

## Introduction

Glioma is the most devastating primary intracranial tumor, characterized by diffuse invasion, induction of neuro degeneration, and resistance to various chemotherapeutic agents (Kang and Adamson, 2015; Babu et al., 2016). Despite the availability of multimodal treatments, the diagnosis and outcomes for glioma patients are still poor, with a median survival time of approximately 14.6 months (Li et al., 2019; Pinel et al., 2019). Hence, elucidating the molecular pathogenic process in gliomagenesis is vital for identify novel therapeutic targets.

The cut-like homeobox 1 (CUX1), also known as CULT1 or CDP, belongs to the family of homeodomain (HD) transcription factors (Liu et al., 1991; Sansregret and Nepveu, 2008; Hulea and Nepveu, 2012). CUX1 shares four similar DNA-binding domain architectures, including a CUT homeodomain and three CUT repeats (CR1, CR2, CR3), which are evolutionarily and functionally conserved from metazoans to humans (Ludlow et al., 1996; Vanden Heuvel et al., 2005; Kedinger et al., 2009). As a major member of the CUX family, it is involved in multiple biological processes including cell differentiation, proliferation, cell cycle regulation, tissue development, and double strand breaks (DSBs) repair response, besides, CUX1 expression was abnormally elevated in many malignant tumors and was implicated in tumorigenesis (Ramdzan and Nepveu, 2014; Wong et al., 2014; Jo et al., 2017). Many lines of evidence have indicated that overexpression of CUX1 was present in various types of cancers, such as melanoma, pancreatic cancer, multiple myeloma, and breast cancer (Fan et al., 2014; Vadnais et al., 2014; Krug et al., 2020). In our previous study, we also found that the CUX1 level was associated with tumor WHO grade and the malignant proliferation index in glioma, which provides additional evidence for the investigation of the CUX1-drived molecular mechanism in gliomagenesis (Wu et al., 2019).

The canonical Wnt/β-catenin signaling pathway was activated in gliomagenesis and involved in proliferation, apoptosis, cell invasiveness and angiogenesis (Wan et al., 2011; Nager et al., 2012). The upregulation of WNT3a and WNT1 in glioma stem cells (GSCs) has been reported in the progression of malignant transformation and gliomagenesis (Liu et al., 2011; Zhang et al., 2011). Besides, the expression of β-catenin, WNT3a and TCF4 were prevalent in astrocytic tumors and the nuclear accumulation of β-catenin was correlated with histological malignancy grade (Sareddy et al., 2009; Kaur et al., 2013). Griesmann et al. 2013 identified WNT5a as a crucial downstream effector of transcription factor CUX1 and CUX1/WNT5a/NFAT axis exerted crucial role in tumorigenesis and drug-resistance in pancreatic cancer (Griesmann et al., 2013). EMT is a complex biological process in which epithelial cells undergo varieties of biochemical changes and finally transformed into mesenchymal phenotype, which exerted a pivotal role in tumor progress, invasion and metastasis (Pastushenko and Blanpain, 2019). There was also evidence to show that the activation of CUX1/WNT signaling regulated epithelial-mesenchymal transition (EMT) in EBV infected epithelial cells (Malizia et al., 2009). Recently, a vitro study has also indicated that by cooperating with GLIS1, CUX1 promoted tumor cell migration and invasion by stimulating TCF/β-catenin transcriptional activity and epithelial-mesenchymal transition (EMT) in breast cancer (Vadnais et al., 2014). Yet, the biological function of CUX1 and it-induced potential molecular mechanism in gliomagenesis were still elusive.

The objective of this study was to investigate the effects of CUX1 expression on physiological functions and the regulatory mechanism of CUX1 gene in the Wnt/β-catenin pathway. Our data provide insight into the pathogenesis, diagnosis, and novel targets of glioma in gene therapy.

## Materials and Methods

### Patients and Specimens

This study included 80 glioma tissue samples and 15 normal brain tissues collected between August 2015 and August 2019 at Linyi People’s Hospital, Shandong, China (Supplementary Tables S1, S2). Patients who had undergone radiotherapy and chemotherapy were excluded from the study. All fresh glioma tumor tissues were immediately transferred to liquid nitrogen and stored at −80°C for subsequent western blotting (WB) and qRT-PCR assays. The remaining tissues were fixed in 4% paraformaldehyde (PFA) for immunohistochemical staining. Each patient signed informed consent to participate in the study. The use of patients’ samples was approved by the Ethics Committee of Linyi People’s Hospital and this study protocol was performed in accordance with the Declaration of Helsinki.

### Reagents and Antibodies

Antibodies for WB, immunohistochemistry (IHC), and immunofluorescence (IF) included CUX1 rabbit mAb (No.11733-1-AP; WB 1:1500; IHC 1:150; IF 1:300), c-Myc rabbit mAb (No.24072-1-AP; WB 1:1000), β-catenin mouse mAb (No.66379-1-Ig; WB 1:4000; IF 1:200), MMP-2 rabbit mAb (No.10373-2-AP; WB 1:3000), MMP-7 rabbit mAb (No.10374-2-AP; WB 1:2000), MMP-9 rabbit mAb (No.27306-2-AP; WB 1:2000), RHOA mouse mAb (No.66733-1-Ig; WB 1:3000), RHOB rabbit mAb (No.14326-1-AP; WB 1:2500), RHOC rabbit mAb (No.10632-1-AP; WB 1:1500), ROCK1 rabbit mAb (No.21850-1- AP; WB 1:2000), CDK2 rabbit mAb (No.10122-1-AP; WB 1:800), CDK4 mAb (No.11026-1-AP; WB 1:3000), CDK6 mAb (No.14052-1-AP; WB 1:1500), CyclinD1 rabbit mAb (No.26939-1-AP; WB 1:1000), CyclinE1 rabbit mAb (No.11554-1-AP; WB 1:800), GAPDH mouse mAb (No.60004-1-Ig; WB 1:3000); all antibodies were purchased from Proteintech (Wuhan, China). Antibodies against Axin2 (No. ET1703-96; WB 1:3000; IF 1:300) were obtained from Huabio Technology (Hangzhou, China). Dulbecco’s Modified Eagle’s Medium (DMEM) and fetal bovine serum (FBS) were purchased from Gibco (Carsbad, CA, United States). The cell cycle kit and CCK-8 kit were obtained from Beyotime (Jiangsu, China).

### Vector Construction and Transfection Assays

To overexpress CUX1, the coding sequence (NM_181552.4) was obtained from the NCBI and was synthesized to facilitate cloning. The synthesized sequence was ligated and subcloned into pBABE lentivirus vector and an empty vector was used as the control group. Three siRNA sequences targeting CUX1 were used: shRNA#1: forward 5′-GCA​UAA​GCU​CAG​UCU​GAA​ATT-3′, reverse 3′-UUU​CAG​ACU​GAG​UUA​UGC​TT-5’; shRNA#2: 5′-GCA​AGG​AGC​CAU​UUC​ACA​ATT-3′, reverse 3′-UUG​UGA​AAU​GGC​UCC​UUG​CTT-5’. Lentiviral particles were purchased from Genechem. Co., Ltd. The siRNAs and lentiviral particles were transfected into cells using Lipofectamine 3000 (Invitrogen, United States) according to the manufacturer’s protocol. Puromycin was used to screen the stable cells, which were collected for subsequent experiments. As a positive control for inhibition of Wnt/β-catenin signal transduction by siRNA, we used siRNA targeting Axin2 with the sequence (forward 5′-GCA​TAG​ATT​GTT​ACT​GCT​A-3′, reverse 3′-GCA​CAG​ATT​ATT​ACT​GCT​A-5′) and siRNA targeting CTNNB1 with the sequence (5′-CCU​UCA​CUC​AAG​AAC​AAG​UTT-3′, reverse 3′-TTG​GAA​GUG​AGU​UCU​UCU​UCA-5′).

### Cell Culture

The cell lines HA 1800, U251, A172, SF295, CRT, TJ905, and PT2 were cultured in DMEM containing penicillin/streptomycin and 10% FBS in a 5% CO_2_ incubator at 37°C. The cells were sub-cultured when cultures reached a confluence of 70–80%; DMEM was replaced every 2–3 days. All glioma cell lines and Normal human astrocyte cells (NHAs) were purchased from Cell Bank, Shanghai Academy of Science, Shanghai, China.

### RNA Extraction and Real-time Quantitative Reverse Transcription PCR

RNA was harvested using the TRIzol reagent (Invitrogen), according to the manufacturer’s guidelines. The RNA was transcribed into cDNA by miscriptreverse transcription kit (Takara, Japan). The level of mRNA was quantified by q-PCR with the QuantiTect SYBR Green PCR kit (Takara, Japan). The reaction conditions were 95°C for 10 min, followed by 95°C for 15 s for 40 cycles, and 60°C for 60 s. Primers used for PCR were as follows: CUX1-forward: 5′-AGC​CGA​AAC​CAT​AGC​TCT​TGA-3′; CUX1-reverse: 5′-GCC​CTT​TCG​AGG​TCC​GTC​AT-3′; GAPDH-forward: 5′- TGC​ACC​ACC​AAC​TGC​TTA​GC-3′; GAPDH-reverse: 5′-GGCA TGC​ACT​GTG​GTC​ATG​AG-3′. The mRNA level of target genes was compared to GAPDH by qPCR using the comparative cycle threshold (2^–ΔΔCT^) method. All assays were independently performed in triplicate.

### Western Blotting Analysis

Expression of target genes was determined by WB as previously described with the following modifications (17). Briefly, samples were lysed by radioimmunoprecipitation assay (RIPA) buffer containing a phosphatase and protease inhibitor cocktail (Beyotime, Jiangsu, China). The supernatants were examined for protein content using the BCA method. Equal amounts of protein were separated by SDS-PAGE gels, then transferred onto PVDF membranes (Millipore, Billerica, MA, United States). After blocking for 1–3 h, the membranes were probed with proper primary and secondary antibodies. Bands were visualized by enhanced chemiluminescence and captured with ultrasensitive chemiluminescence imaging system. Protein bands were quantified by using the ImageJ software version 1.8.0 (NIH, US) and all experiments were independently performed in triplicate.

### Immunofluorescence Analysis and Immunohistochemical Staining

For IF assays, cells were grown on coverslips and were fixed in 4% PFA on ice for 20 min. After washing twice with phosphate-buffered saline (PBS), cells were permeabilized with TritonX-100 (Sigma) for 15 min. Cells were blocked with 3% goat serum and 2% bovine serum albumin (BSA), then incubated with β-catenin antibody and Axin2 antibody at 4°C overnight. Subsequently, coverslips were incubated with TRITC-conjugated anti-rabbit secondary antibody after washing twice with PBS at room temperature for 40 min. Then, coverslips were washed again with PBS and incubated with DAPI at 37°C for 30 min. Fluorescence was monitored by Olympus inverted confocal microscope. The semi-quantitative fluorescence intensity analysis of different cell compartments was assessed by ImageJ software. Immunohistochemistry assay was performed with avidin-biotin immunoperoxidase technique as described previously (Wu et al., 2019). CUX1 antibody was used at a dilution of 1:3000. Staining results were scored according to the following standards: The stained sample was assigned a combined IHC score (0–6), which was obtained by adding the intensity score (0–3) and the percentage score (0–100%). Each combined score is shown (0 [negative], score 2 [++, <10%], score 3 [+, >50%], score 4 [++, <50%], score 5 [++, >50%], score 6 [+++,100%]). We defined samples with scores <4 as the low CUX1 expression group and scores ≥4 as the high CUX1 expression group.

### Cell Proliferation Assays

For the colony formation assay, transfected cells were seeded in 6-well plates (500 cells/well) and incubated with 10% FBS for 2 weeks at 37°C for 2 weeks, then fixed with 4% PFA. After washing with PBS, cells were strained with 0.1% crystal violet (Beyotime) for 30 min. The colony forming efficiency was monitored under a microscope. This assay was performed in triplicate. Cell viability was also assessed using the cell counting kit 8 (CCK-8, Beyotime) method at 0, 24, 48, and 72 h. According to the manufacturer’s instructions, cells were seeded in 96-well plate (2000 cells/well). Each well contained 100 μl DMEM and 10 μl CCK-8. The culture plate was incubated at room temperature for 3 h. Absorbance of each well at 450 nm was measured using the Synergy Microplate Reader (Biotek). All assays were independently performed in triplicate.

### Flow Cytometry Assay

For cell cycle analysis, glioma cells were harvested at 48 h post-transfection by trypsinization and fixed with pre-cooled 70% ethanol at −20 °C overnight. After being centrifuged at 1000 × g for 5 min, the cells were stained with 400 µl propidium iodide (PI) and 100 µl RNase A (Beyotime, Shanghai, China) at 37°C for 30 min. A total of 10,000 cells per sample were analyzed by flow cytometry (BD, Bioscience, CA, United States), and the cell-cycle populations were determined by ModFit software. All assays were independently performed in triplicate.

### Transwell Assay and Wound Healing Assay

For the assessment of cell invasion, approximately 5×10^4^ cells in 100 µl DMEM medium without FBS were placed in the upper Matrigel chamber (BD, Bioscience, United States). A volume of 500 µl DMEM with 10% FBS was added to the lower chamber. Next, 10 μg/ml mitomycin was added to the DMEM to repress cell proliferation to greatest extent. After incubation for 24 h at 37°C in 5% CO_2_ atmosphere, the cells on the surface of the insert were removed using a cotton swab. Then, glioma cells that passed through the membrane were stained with 1% crystal violet and counted under a microscope.

For the wound healing assay, 5×10^5^ transfected glioma cells were cultured in 6-well plates and incubated until the cells grew to 90% confluence. The confluence plates were scratched with a P-10 pipette tip, washed with PBS to remove the detached cells and cultured in serum-free medium supplemented with 10 μg/ml mitomycin. The wound-closing procedure was observed under a microscope at 0, 24, and 48 h, respectively. All assays were independently performed in triplicate.

### Gene Expression Profile Data Acquisition

Microarray RNA-seq data of glioma patients were downloaded from the GEO database (https://www.ncbi.nlm.nih.gov/geo/) under the accession number GSE16011, GSE51006, GSE67089, GSE45921, GSE7696, and GSE4290. The GPL8542 platform (Affymetrix Gene Chip Human Genome U133 Plus 2.0 Array) was used. The clinical data and related RNA-seq data from patients with glioma were obtained from TCGA database (https://xenabrowser.net/datapages/). Data for co-expression analysis were obtained from the CGGA (http://www.cgga.org.cn/), GTEx and CCLE database (https://xenabrowser.net/datapages/).

### Bioinformatics Analysis

Gene set enrichment analysis (GSEA) was performed to identify the essential biological pathways that were significantly enriched in the CUX1^high^ group. The enrichment status estimates of CUX1-associated signaling pathways were also obtained using the gene set variation analysis (GSVA) package. Other R packages, “ggpubr”, “ggplot2”, “limma”, “dplyr”, and “tidyr” were also applied to visualize the results of bioinformatics analysis.

### Statistical Analysis

Statistical analysis was performed using Origin2018 and Sigmaplot version 14.0 for Windows. All data were obtained from three independent assays and presented in the form of Mean ± SD. The Student’s *t*-test was used to analyze differences between two groups. Comparisons between more than three groups were determined using one-way ANOVA analysis of variance followed by the Turkey post-hoc test. The χ^2^test examined the relationship between CUX1 expression and clinicopathological characteristics. Kaplan-Meier survival analysis and the log-rank test were used to investigate the overall survival (OS). A *p*-value <0.05 was considered statistically significant.

## Results

### Expression of Homeobox Cut Like 1 in Glioma Was Associated With Poor Prognosis

**FIGURE 1:**
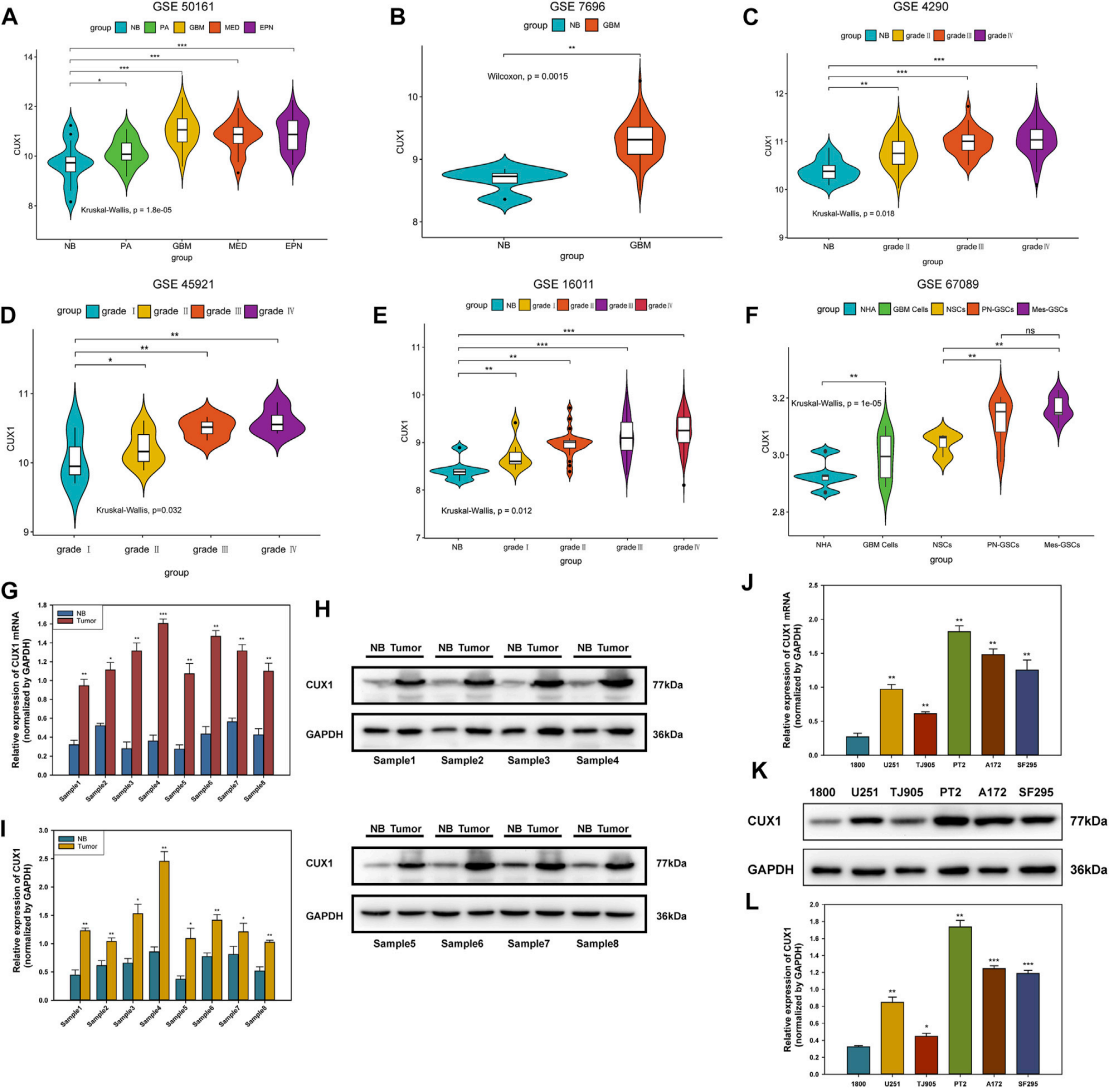
CUX1 expression was upregulated in glioma. **(A)** Association between the expression of CUX1mRNA with various subtypes of intracranical tumors available on the GEO database (GSE501161). **(B–E)** Correlation between the CUX1mRNA level and WHO grades of glioma. Increased expression of CUX1mRNA was detected with pathological grade rising, based on analysis of RNA-seq data from GSE7696, GSE4290, GSE45921, and GSE16011. **(F)** Expression patterns of CUX1mRNA in normal human astrocyte (NHA) and glioblastoma (GBM) cells as well as their progenitor cells (NSCs and GSCs). **(G)** The expression levels of CUX1mRNA in fresh glioma tissues and adjacent non-tumor tissues were analyzed by RT-qPCR. **(H,I)** Western blotting analysis showing the levels of CUX1 protein and mRNA in fresh glioma tissues and adjacent noncancerous tissues among eight groups of samples. **(J**–**L)** qRT-PCR and Western blot analysis showed the expression of CUX1 mRNA and protein in normal astrocyte (HA 1800) and five glioma cell lines (U251, TJ905, PT2, A172 and SF295). (*p < 0.05, **p < 0.01, ***p < 0.001).

**FIGURE 2:**
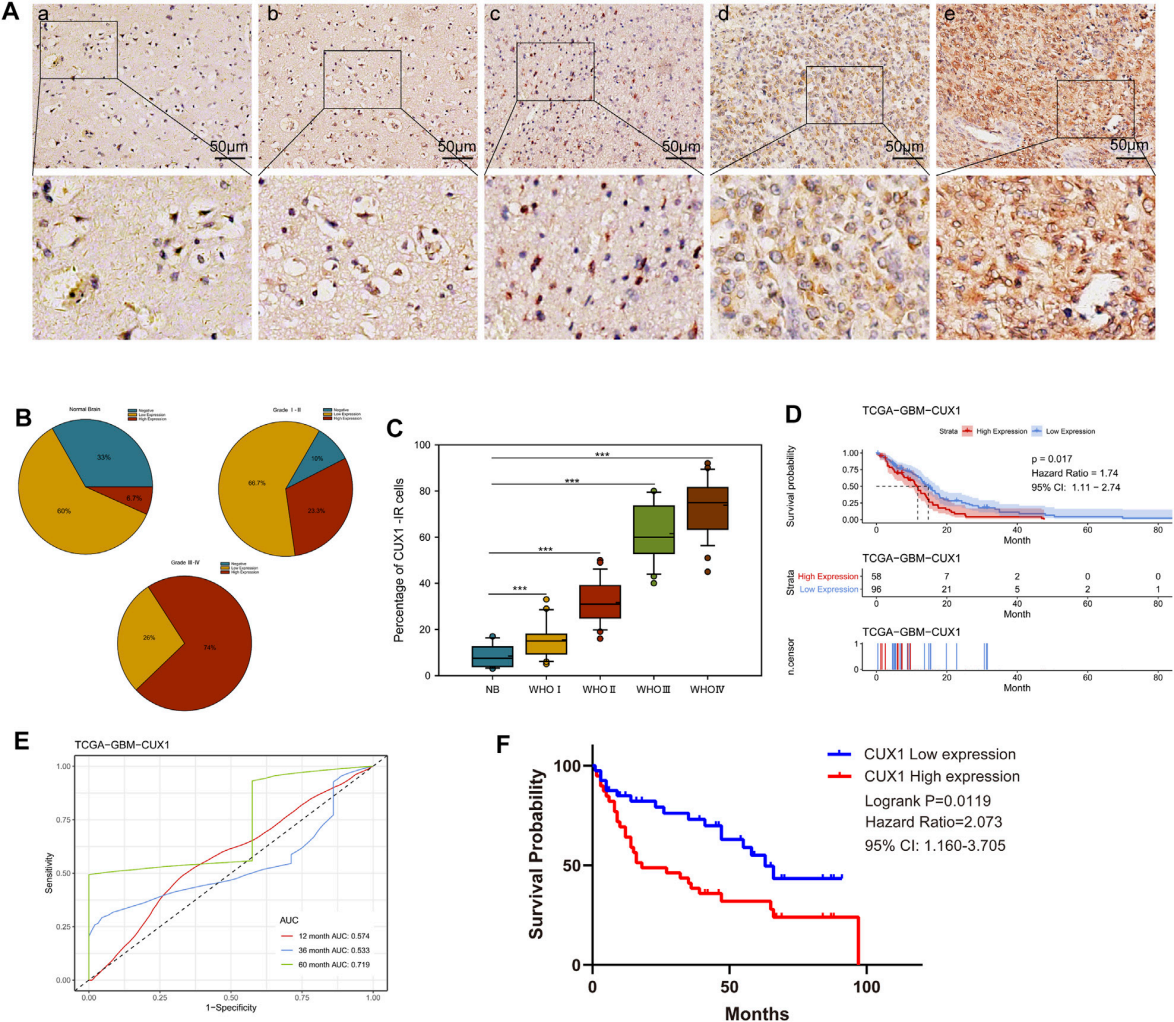
High CUX1 expression predicted poor prognosis in patients with glioma. **(A–C)** The expression levels of CUX1 in 80 glioma tissues and 15 normal brain tissues, measured by immunohistochemistry (IHC). **(A)** Representative images for CUX1 staining in nontumor **(a)** brain samples and WHO grade Ⅰ-Ⅳ glioma tissues **(b–e)**. IHC-stained sections, the scale bar corresponds to 50 μm. **(B)** Statistical analysis of CUX1 expression in normal brain tissue and different clinical grade glioma specimens. **(C)** Quantitative analysis of cell counts showing that the percentage of CUX1-immunoreactivity (IR) cells was significantly increased in glioma tissues compared with nontumor brain tissues. **(D)** Kaplan Meier (KM) curve comparing overall of glioblastoma patients according to expression of CUX1 in TCGA database. Log-rank test. **(E)** Receiver operator characteristic curve analysis of CUX1. AUC, Area under the curve. **(F)** KM-plot of CUX1 expression in patients with glioma based on clinical information data from our study. (*p < 0.05, **p < 0.01, ***p < 0.001).

We initially analyzed the mRNA levels of CUX1 in several types of intracranial tumors and found that the expression of CUX1 mRNA was higher in glioma (GSE50161, Figure 1A). To identify the CUX1 expression pattern in clinical glioma, we analyzed the RNA-seq data using the microarray GSE7696, GSE4290, GSE45921, and GSE16011 from GEO database. The statistical results of the four datasets indicated that the level of CUX1mRNA was significantly upregulated in glioma compared to normal brain tissues (Figures 1B–E). The astrocyte and glioma cells were differentiated from neuro stem cells (NSCs) and glioma stem cells (GSCs), respectively. NSCs, as the most active cells in central nervous system (CNS), are in a state of continuous proliferation and division and are prone to mutation. There are growing evidence to support the possibility that GSCs are derived from the accumulation of mutations in NSCs and astrocyte progentiors (Guelfi et al., 2016). Zong et al. 2015 also reported that both astrocyte/oligodendrocytes and their progenitors (NSCs) could serve as the original cells of glioma (Zong et al., 2015). In the microarray GSE67089, CUX1mRNA was significantly overexpressed in GSCs and GBM cells compared with NHA and NSCs, which suggested that CUX1 may exert a crucial role in gliomagenesis (Figure 1F). Consistent with the findings in the RNA-seq datasets, the results of qRT-PCR and WB assays showed that CUX1 mRNA and proteins were significantly increased in glioma tissues compared to normal brain tissues (Figures 1G–I). Moreover, the expression of CUX1 mRNA and protein in the normal human astrocyte cell line (HA 1800) and several glioma cell lines (U251, TJ905, PT2, A172 and SF295) were detected by qRT-PCR and WB assays. The results showed that CUX1 expression was higher in glioma cell lines (especially PT2 and A172) compared to HA1800 cells (*p* < 0.05, Figures 1J–L). In addition, we reviewed the clinical characteristics of 80 patients with glioma. The IHC assay revealed that CUX1 was mainly located in the cytoplasm/cytomembrane of glioma cells and CUX1 levels were upregulated with increasing World Health Organization (WHO) grade in the glioma samples. (Figures 2A–C, Supplementary Tables S1, S2). To evaluate the prognostic value of CUX1 in glioblastoma patients, the clinical information of 577 patients was obtained from the TCGA RNA-seq database. After dividing the cases into two equal strata according to CUX1 expression, Kaplan-Meier survival analysis revealed that CUX1 overexpression was correlated with shorter OS compared to patients with negative or low CUX1 expression, which was also in line with the results of the survival analysis in our study (Figures 2D,F). Meanwhile, in the Receiver operator characteristic (ROC) curve analysis, the area under curve (AUC) for CUX1 expression in predicting the 60 months survival was 71.9% (Figure 2E). The findings mentioned above revealed that CUX1 served as a negative prognostic factor in patients with glioma.

### Effects of Knockdown and Overexpression of Homeobox Cut Like 1 on Cell Proliferation and Cell Cycle Progression in Glioma Cells

**FIGURE 3:**
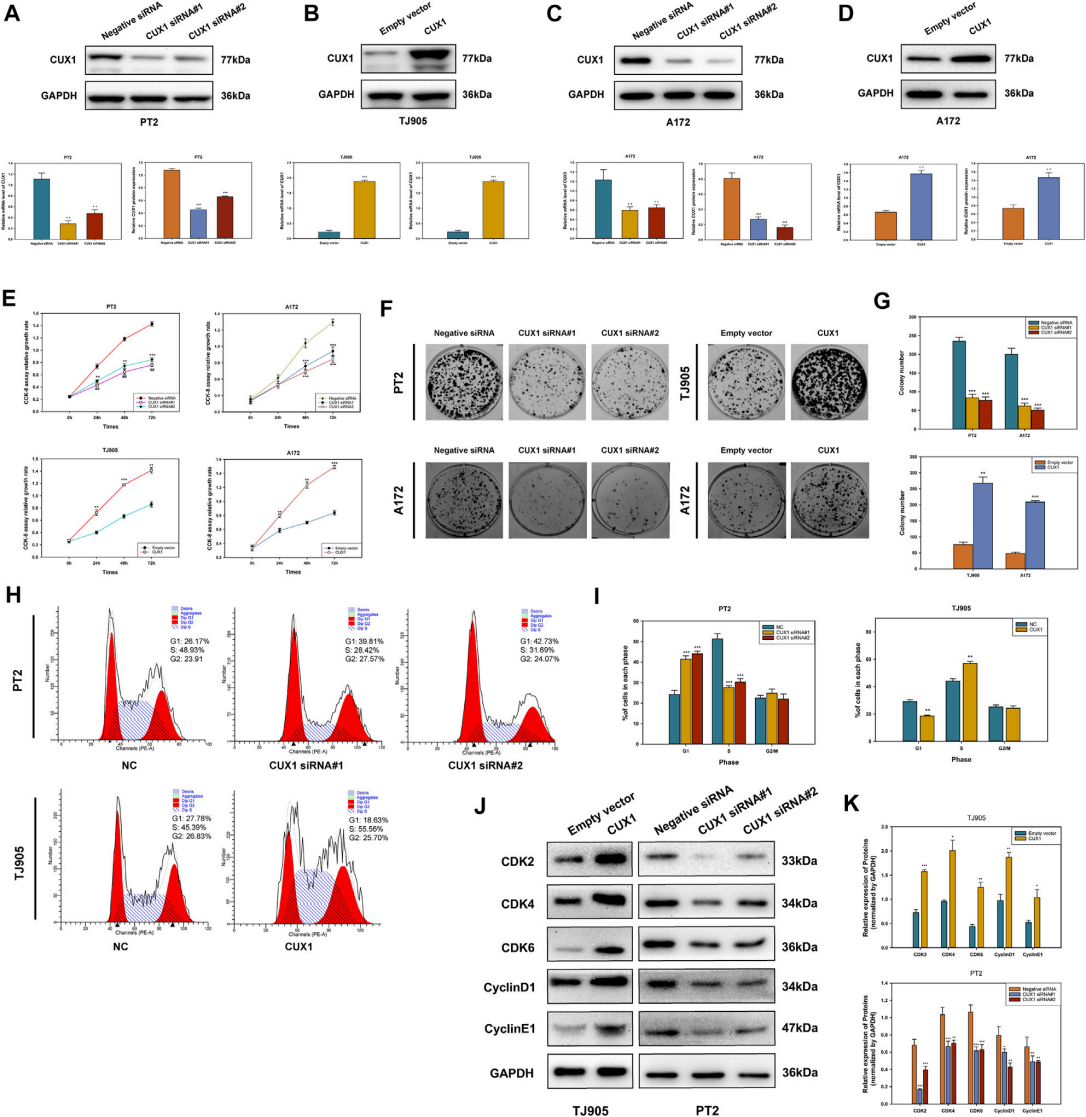
Effects of knockdown and overexpression of CUX1 on cell proliferation and cell cycle progression in glioma cells. **(A)** Transfected with CUX1 siRNA#1 and siRNA#2 decreased the CUX1 protein and mRNA level compared to negative siRNA in PT2 cells. **(B)** Transfected with CUX1 plasmid upregulated the expression of CUX1 protein compared to empty vector in TJ905 cells. **(C,D)** The interference effects of TROAP siRNA and overexpression plasmid in A172 cells. **(E)** Proliferation in PT2, TJ905 and A172 cells was detected by CCK8 assay. **(F,G)** The effect of CUX1 on PT2, TJ905 and A172 cells proliferation was measured by the colony formation assay. **(H,I)** Flow cytometry presented CUX1 induced cell cycle arrest in PT2 and TJ905 cell lines. **(J,K)** Western blot showed the expression of relevant cell cycle proteins in PT2 and TJ905 cells. (*p < 0.05, **p < 0.01, ***p < 0.001).

The gain-of-function and loss-of-function assays were performed to investigate the role of CUX1 in PT2 and TJ905 cell lines. Transfected cells were detected for CUX1 at the protein by WB analysis. The results demonstrated that the expression of CUX1 was significantly lower following transfection with two CUX1-siRNA vectors in PT2 cells, and specifically following transfection with CUX1-siRNA#1 (*p* < 0.05, Figure 3A). Conversely, transfection with the CUX1 plasmid markedly increased the level of CUX1 protein and mRNA compared to transfection with empty vector in TJ905 cells (*p* < 0.05, Figure 3B). Different glioma cell lines exhibit different phenotypes and properties. To eliminate potential interference by different cell line phenotypes, we also selected the A172 cell line for knockdown and overexpression transfection assays, followed by a series of cellular function assays (*p* < 0.05, Figures 3C,D). The effects of CUX1 on glioma cell proliferation were examined by preforming CCK-8 and colony formation assays. The results indicated that CUX1 overexpression significantly enhanced viability and colony formation in TJ905-trasfected cells compared to control cells, while CUX1 knockdown significantly decreased proliferation of PT2 cells. These observations were also consistent with the results of parallel CCK8 and colony formation assays in the A172 cell line (*p* < 0.05, Figures 3E–G). Next, the role of CUX1 in cell cycle progression was further explored *via* flow cytometry analysis. The findings showed that there were more TJ905 cells in the S phase and less in the G1 phase following transfection with the CUX1 overexpression vector; whereas, transfection of CUX1-siRNA resulted in a higher proportion of PT2 cells in the G1 stage (*p* < 0.05, Figures 3H,I). Similarly, the effects of CUX1 expression on cell cycle proteins were also detected by WB analysis. A higher expressions of Cyclin D1, Cyclin E1, CDK2, CDK4 and CDK6 were observed in TJ905 cells presenting CUX1 overexpression compared to controls, and conversely, these proteins were all down-regulated following CUX1-siRNA treatment (*p* < 0.05, Figures 3J,K).

### Homeobox Cut Like 1 Promoted Invasion and Migration in Glioma Cells

**FIGURE 4:**
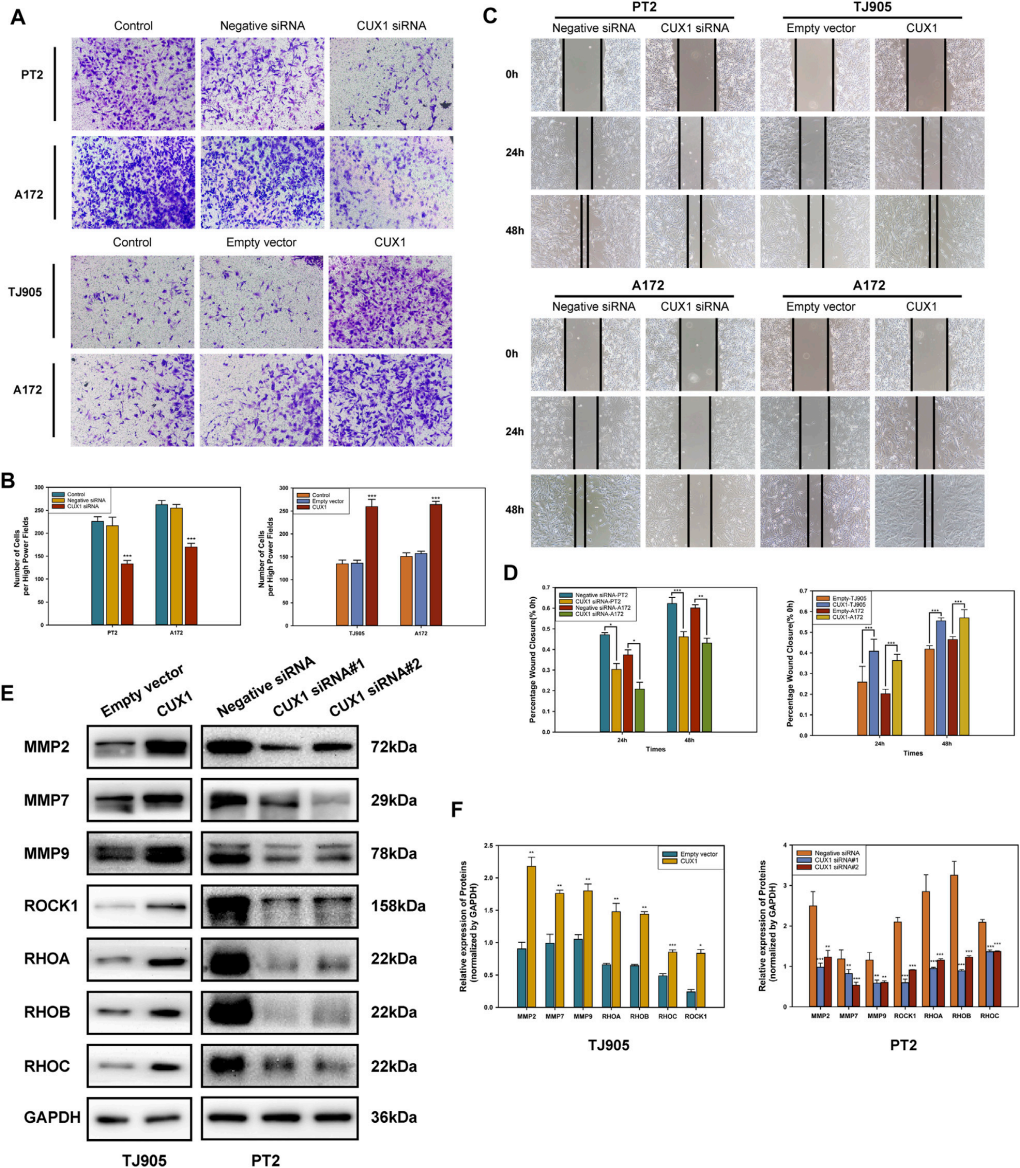
Effects of overexpression and knockdown of CUX1 on the migration and invasion of glioma cells. **(A,B)** Transwell assay showed the invasion of TJ905, PT2 and A172 cells after transfection with CUX1 siRNA or CUX1 overexpression plasmid (magnification ×200). **(C,D)** Wound healing assay was used to detect the cell migration ability after overexpression or knockdown of CUX1 after 24 and 48 h in TJ905, PT2 and A172 cells (magnification ×200). **(E,F)** Western blot analysis showed the change of migration-related proteins in PT2 and TJ905 cells.

The roles of CUX1 in regulating cell invasion and migration ability were investigated using Transwell invasion and wound healing assays. Our findings indicated that the invasive and migrative abilities of TJ905 cells had increased following transfection with CUX1 overexpression plasmid, and this ability was inhibited following transfection with CUX1-siRNA. Similarly, A172 glioma cells also exhibited enhanced migration and invasion capability when treated with the CUX1 overexpression plasmid compared to the empty vector, while attenuated migration was observed following transfection with CUX1 siRNA (*p* < 0.05, Figures 4A–D). WB analysis was then performed to assess the effects of CUX1 overexpression on migration-associated genes. Knockdown of CUX1 significantly down-regulated the expression of MMP2, MMP7, MMP9, ROCK1, RHOA, RHOB, and RHOC protein in PT2 cells compared to control, while the respective gene levels were dramatically elevated in TJ905 cells (Figures 4E,F). These data suggested that CUX1 promoted glioma infiltration and spread by regulating the expression of migration-related proteins.

### The Downregulation of Homeobox Cut Like 1 Reversed the Epithelial-Mesenchymal Transition

**FIGURE 5:**
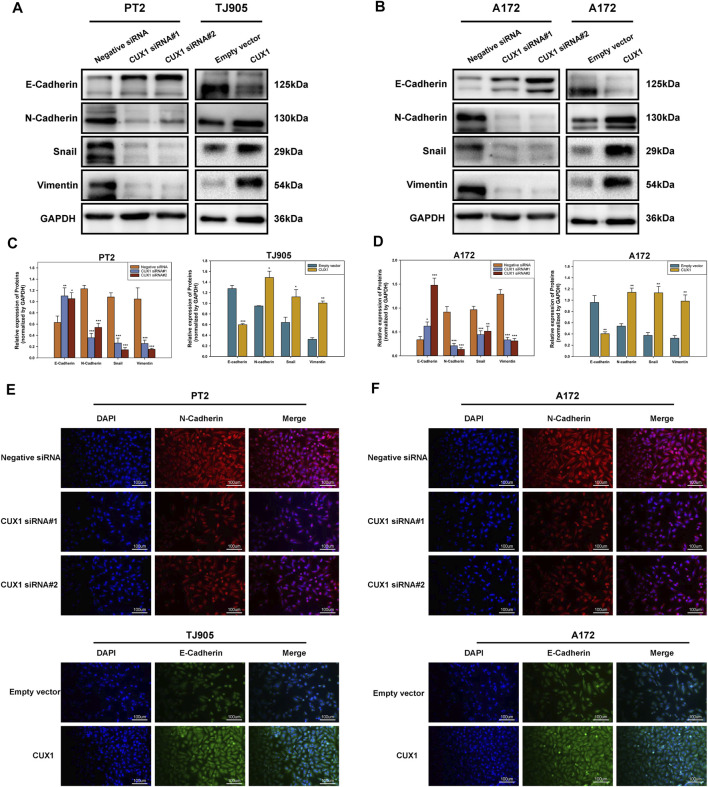
The downregulation of CUX1 reversed the epithelial-mesenchymal transition (EMT). **(A–D)** Western blotting showing the decreased protein levels of mesenchymal marker (N-cadherin, vimentin and snail) and the increased protein levels of epithelial marker (E-cadherin) after knockdown of CUX1 in PT2 cells, while conversed phenomenon was presented in TJ905 following CUX1 overexpression treatment. The same effect was observed after treatment with CUX1-siRNA/CUX1 plasmid in A172 cell line. Representative images were presented in **A** and **B**, and quantitative analysis were shown in **C** and **D**. **(E,F)** Immunofluorescence staining showing the same outcomes as for CUX1 in PT2, TJ905 and A172 cells after siRNA treatment and overexpression plasmid treatment. The scalebar corresponds to 100 μm. (*p < 0.05, **p < 0.01, ***p < 0.001).

The epithelial-mesenchymal transition (EMT) is a complex process that enable epithelial cells lose cell adhesiveness/polarity and then transform into the mesenchymal cells, which have an increased invasive and metastatic ability and gain the malignant phenotype. Moreover, EMT is further correlated with increased activities of matrix metalloproteins (MMPs), which facilities cell invasion *via* degrading extracellular matrix proteins. Hence, we next used WB analysis to measure the effects of CUX1 downregulation/upregulation on the activities of EMT markers in glioma cell lines. The results of WB assay showed that levels of mesenchymal markers (N-cadherin, Snail and Vimentin) decreased in PT2 and A172 cells following CUX1-siRNA transfection, while, the expression of epithelial adhesion molecular E-cadherin was upregulated (*p* < 0.05). After transfecting with CUX1 overexpression vector in TJ905 and A172cells, WB assay demonstrated a decline of E-cadherin protein levels, but the expression levels of N-cadherin, snail and vimentin were obviously upregulated (*p* < 0.05, Figures 5A–D). Immunofluorescence staining assays provided further confirmation that the effect of CUX1 on the epithelial-mesenchymal transition in PT2, TJ905 and A172 glioma cell lines (*p* < 0.05, Figures 5E,F). Taken together, these results suggested that the CUX1 overexpression promoted the epithelial-mesenchymal transition, while, downregulation of CUX1 could reverse the malignant phenotype of glioma.

### Homeobox Cut Like 1 Promoted the Malignant Phenotype by Activating Wnt/β-Catenin Signaling

**FIGURE 6:**
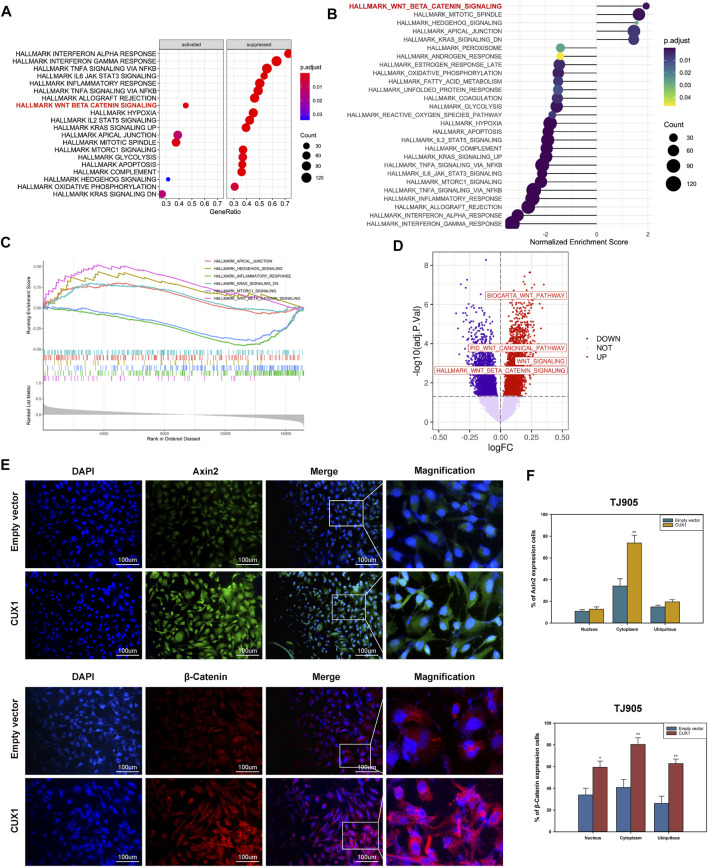
Overexpression of CUX1 enhanced the activity of Wnt/β catenin signaling pathway in glioma cells. **(A–C)** Gene set enrichment analysis (GSEA) pathways enriched by CUX1. **(A)** The dotplot of partially enriched pathway. **(B)** The dotplot of the up- and down regulated pathways. **(C)** The up-regulated pathway of Wnt/β-catenin. **(D)** Wnt/β-catenin pathway was enriched and upregulated in glioma by gene set variation analysis (GSVA) analysis. **(E,F)** Immunoflurescence showed that overexpression of CUX1 increased expression of Axin2 proteins in cytoplasm of TJ905 cells compared to empty group. Meanwhile, CUX1 overexpression also increased the expression of β-catenin in nucleus and cytoplasm of TJ905 cells compared to control.

**FIGURE 7:**
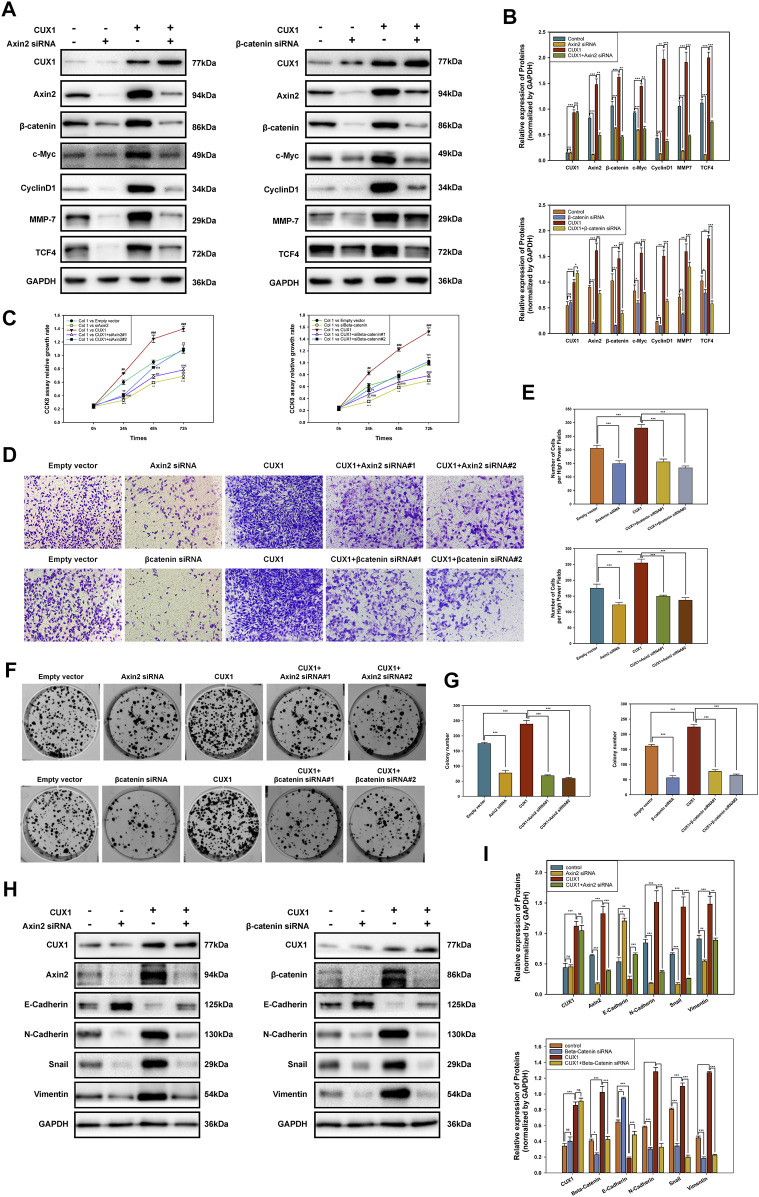
Inhibiting Axin2 or β-catenin could partially reverse the malignant phenotype caused by CUX1 overexpression in gliomagenesis. **(A,B)** Western blot analysis presented the levels of subunits in Wnt/β-catenin signaling pathway after knocking-down Axin2 or β-catenin in TJ905 cells ectopically overexpressing CUX1. **(C)** CCK8 assay was used to detect the change of proliferation capability after inhibiting Wnt/β-catenin in TJ905 cell lines. *, compared with the empty vector group; #, compared with the empty vector group; α, compared with the empty vector group; γ, compared with the empty vector group. **(D,E)** Transwell assay to evaluate the reversal effect of silencing Wnt/β-catenin pathway on the migration activity in TJ905 cell lines. **(F,G)** The change of proliferation capability after inhibiting Wnt/β-catenin was detected by colony formation assays in TJ905 cell lines. **(H,I)** Western blot analysis presented the levels of EMT markers (N-cadherin, E-cadherin, Vimentin and Snail) after inhibiting Wnt/β-catenin pathway (knocking-down Axin2 or β-catenin) in TJ905 cells ectopically overexpressing CUX1.

**FIGURE 8:**
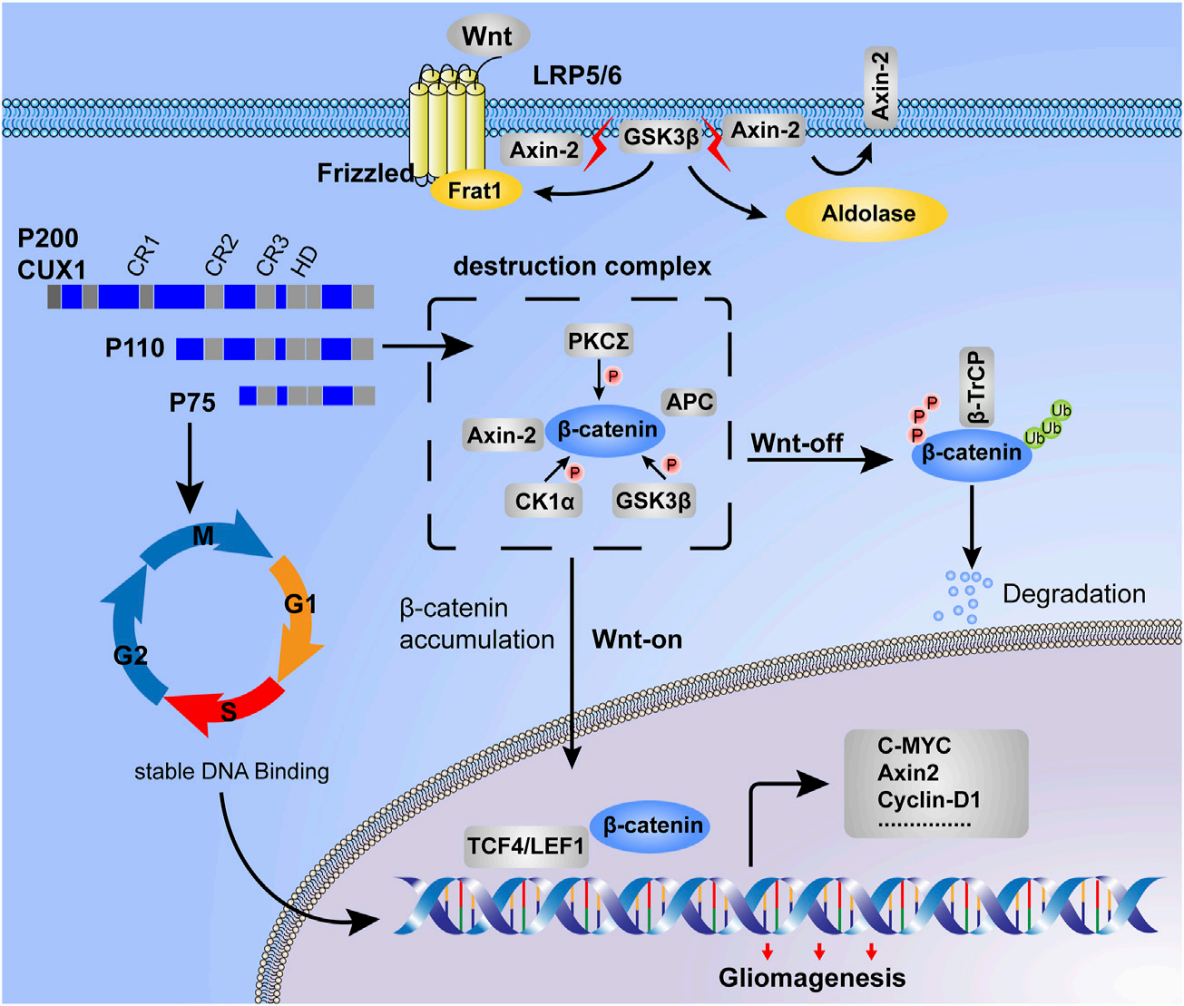
Working model for CUX1-induced malignant phenotype in gliomagenesis. Destruction complex was the essential step for β-catenin phosphorylation, meanwhile, the phosphorylated status of β-catenin was determined for its stability. In the Wnt-off state, β-catenin was recruited by the destruction complex and induced degradation in proteasome. However, in the state of Wnt-on, due to the disassembly of the destruction complex, β-catenin was accumulated in cytoplasm and then translocated into the nucleus. On the one hand, DNA-binding dynamics of CUX1 showed a cell cycle-dependent manner, our study found that glioma cells with CUX1 overexpression represented the accelerated entry into S phase and cell proliferation. On the other hand, we also found that transcription factor CUX1 could upregulate the expression of Axin2/β-catenin and activated Wnt/β-catenin signaling pathway to promoted the transcription of downstreamed cell motility-related genes (such as CyclinD1, C-Myc and MMP7) and progress of epithelial-mesenchymal transition (EMT), inducing the malignant phenotype during gliomagenesis.

Based on GSEA enrichment analysis, we identified 30 CUX1-associated significantly enriched pathways (adj.*p*.value < 0.05), including 11 activated pathways (normalized enrichment score [NES] >0) and 19 inactivated pathways (NES<0). The results, as shown in Figures 6A–C, indicated that the Wnt/β-Catenin pathway was activated by CUX1, which was also confirmed by GSVA analysis (*p* < 0.05, Figure 6D). Moreover, the transcription factor CUX1, by targeting key subunits of the Wnt/β-Catenin pathway, showed a positive correlation with mRNA expression of Axin2, CTNNB1, and TCF4 in most normal and cancer tissues or cell lines (Supplementary Figure S1A–E). The interaction network of CUX1 protein was analyzed using the GeneMANIA database, which suggested that CUX1 and several key regulators of the Wnt/β-catenin signaling were co-expressed (Supplementary Figure S1F–G). To confirm the bioinformatics results, a series of functional assays were performed to explore the potential molecular mechanism involved in CUX1-induced gliomagenesis. Axin2 and β-catenin (CTNNB1) serve as crucial regulatory factors involved in Wnt/β-catenin signaling. The IF assays revealed that CUX1 overexpression significantly induced substantial accumulation of Axin2 in cytoplasm and β-catenin in the nucleus and cytoplasm of TJ905 cells compared to controls (*p* < 0.05, Figures 6E,F). In addition, the WB analysis showed that when compared with control group, cells transfected with the CUX1 overexpression vector significantly increased the expression of downstream genes involved in Wnt/β-catenin signaling, such as CyclinD1, C-Myc, MMP7, and TCF4 (*p* < 0.05, Figures 7A,B). While the expression of the respective proteins and the CUX1-induced tumor-promoting effects (tumor cell proliferation and invasion) were significantly reversed by silencing Axin2 or β-Catenin expression (*p* < 0.05, Figures 7C–G). Importantly, Western blot assay also found the expression levels of EMT markers (N-cadherin, E-cadherin, Vimentin and Snail) were reversed after inhibiting Wnt/β-catenin pathway (knocking-down Axin2 or β-catenin) in TJ905 cells ectopically overexpressing CUX1 (*p* < 0.05, Figures 7H,I). Taken together, our findings indicated that CUX1 induced a malignant phenotype and tumorigenesis, at least in part *via* activation of the Wnt/β-Catenin signaling pathway (Figure 8).

## Discussion

Gliomas, a CNS tumor, comprise approximately 80% of all primary malignant brain tumors (Shaver et al., 2019). Due to the multifocal tumor infiltration and spreading, the outcomes for these patients remain poor and the 5 years OS of glioblastoma patients is less than 5% (Falkenstein et al., 2020; Stylli, 2020). It is widely accepted that gliomagenesis is likely to involve cell immortalization, abnormal activation of signaling pathways, and multiple gene mutations. Hence, gene therapy has become a novel therapeutic option for glioma.

The present study examined the molecular mechanisms and physiological functions of CUX1 in glioma. When analyzing datasets from the GEO and TCGA RNA-seq databases, the findings indicated that CUX1 was overexpressed in glioma tissues and exhibited a significant association with shorter OS, which was also confirmed with our clinical analysis results. Furthermore, our previous study also indicated that CUX1 overexpression was significantly correlated with several clinicopathological features, such as P53 mutation, Ki67 expression, and WHO tumor grade (Wu et al., 2019). These results indicated that CUX1 had prognostic value in patients with glioma. CUX1 is widely expressed in all metazoans and is functionally conserved in human tissues (Liu et al., 2020). In accordance with our results, recent studies have reported that CUX1 overexpression was significantly associated with tumorigenesis and poor prognosis in patients with multiple myeloma, uterine leiomyomas, colorectal cancer, and high-grade breast cancer (Moon et al., 2002; Cancer Genome Atlas, 2012; Vadnais et al., 2014). Kühnemuth et al. reported that CUX1 promoted tumor-associated macrophages (TAM) to present M2 polarization-dependent angiogenesis and tumorigenesis *via* inhibiting binding of NF-κB P65 to the promoter region of the chemokine CXCL10 in pancreatic ductal adenocarcinoma (Kuhnemuth et al., 2015). Kojima et al. 2011 also found a complex regulatory cascade involving CUX1, which activated miR-24 to repress AFP gene expression *via* inhibition of the ZBTB20 transcription factor, suggesting that aberrantly elevated CUX1 expression was correlated with tumorigenesis in hepatocarcinoma (Kojima et al., 2011). A genome-wide location analysis revealed that the biochemical functions and expression of CUX1 were regulated in a cell cycle-dependent manner. The level of histone nuclear factor D (HiNF-D), which is a DNA-binding partner of CUX1, was elevated and accompanied Cdc25A phosphorylation, whereas, DNA-binding activity was attenuated in early G1 and G2 phases in normal cells (Sansregret et al., 2010; Sansregret and Nepveu, 2011). Moreover, mouse embryonic fibroblasts (MEFs) from CUX1^−/−^ mice proliferated more slowly and presented cells having a longer G1 phase, whereas, cells with CUX1 overexpression showed accelerated entry into S phase, and stimulated proliferation in many cell types (Sansregret et al., 2006). In line with these findings, the present study revealed that CUX1 overexpression induced cell accumulation in S phase and increase the expression of cell cycle-related proteins, including CDK2, CDK4, CDK6, and Cyclin E and Cyclin D1. To the authors’ knowledge, this was the first *in vitro* study to report that CUX1 promoted proliferation by activating G1/S transition in glioma cells.

Tumorigenesis is a multistep and complex process that involves adhesion and degradation of extracellular matrix components and the active migration from the primary tumor (Panciera et al., 2020). Our study found that CUX1 overexpression significantly increased the invasion and migration of glioma cells. Conversely, silencing CUX1 showed significantly retarded capability of infiltration and spread in glioma cells, while, decreasing the expression of migration-related genes. Recent studies indicated that even non-pure epithelial cells can switch from non-polar epithelial phenotype into mesenchymal phenotype, and the existence of this phenomenon was also confirmed in different experimental models, referred to as EMT-like process (Tam and Weinberg, 2013; Iser et al., 2017). Iser et al. 2017 reported that reactive astrocytes might undergo EMT-like under stimuli of GBM cells. As a result, the positive feedback of astrocytes and glioma cells could induce cell migration and invasion to support gliomagenesis (Iser et al., 2019). Our data showed that knockdown of CUX1 suppressed the expression levels of mesenchymal markers (N-cadherin, vimentin and snail), and enhanced the expression of epithelial marker E-cadherin. CUX1 overexpression presented the opposite effects, which indicated that CUX1 participated in the regulation of EMT-like process in glioma. These findings above implied that CUX1 exerted invasion, migration and malignant phenotype-promoting effects on glioma cells.

The Wnt/β-catenin pathway plays a crucial role in various physiological activities, meanwhile, aberrant mutation and activation of Wnt/β-catenin signaling is linked with the occurrence and development of glioma (He et al., 2019; Ma et al., 2019; Chen et al., 2020). β-catenin, as the major subunit of the canonical Wnt pathway, accumulates in cytoplasm and then is transported into the nucleus upon genetic mutations or activation of Wnt, where it binds with T-cell factor/lymphoid enhancer-binding factor (TCF/LEF) to promote the transcription of downstream cell motility-related genes, such as Cyclin D1, C-Myc, MMP7, and TCF4 (Gekas et al., 2016; Mao et al., 2017; Shang et al., 2017). To elucidate the molecular mechanisms underlying CUX1 in gliomagenesis, we performed single gene GSEA analysis using the KEGG curated geneset; the “Hallmark WNT Beta Catenin signaling” was among the top positively enriched pathways in the CUX1 high-expression group. A positive correlation of CUX1 with key subunits of the Wnt/β-catenin pathway was also identified in GTEx, TCGA, CGGA and CCLE datasets. It is well-known that the canonical Wnt pathway is mediated by β-catenin, hence, we speculated that the CUX1-induced gliomagenesis was correlated with the activation of Wnt/β-catenin signaling pathway.

In the present of Wnt ligands (On State), Wnt binds both LRP5/6 and Fzd receptor to initiate phosphorylation of the Axin2/APC/GSK3β complex (destruction complex). Then, β-catenin phosphorylation is inhibited, which prevents its degradation in the proteasome. β-catenin accumulates in the cytosol and then translocates to start the Wnt response gene transcription (Zhang et al., 2012; Stamos and Weis, 2013; He et al., 2019). Our IF assays revealed that CUX1 overexpression induced accumulation of Axin2 in cytoplasm and β-catenin in nucleus and cytoplasm, which was in accordance with the notion that the degradation of β-catenin was controlled by Axin2/APC/GSK3β complex in cytoplasm. We hypothesized that aberrantly upregulated CUX1 might prevent β-Catenin from being degraded by Axin2 complex, leading to the substantial accumulation of β-Catenin in cytoplasm and subsequent transportation into the nucleus, where it increased the transcription of downstream genes involved in the activation of Wnt/β-Catenin signaling, ultimately inducing tumorigenesis. As expected, our WB results indicated that CUX1 overexpression increased the level of a series of downstream genes belonging the Wnt/β-catenin pathway such as Cyclin D1, C-Myc, MMP7, and TCF4. The expression of these proteins and the CUX1-induced tumor-promoting effect could be reversed by silencing Axin2 or β-catenin expression. Importantly, WB assay also found the levels of EMT markers (N-cadherin, E-cadherin, vimentin and snail) were reversed after inhibiting Wnt/β-catenin pathway (knocking-down Axin2 or β-catenin) in TJ905 cells ectopically overexpressing CUX1 (*p* < 0.05, Figures 7H,I). It is evidence that Wnt/β-catenin pathway participated the CUX1-induced malignant phenotype in glioma. The findings above were consistent with our hypothesis, confirming that CUX1 was a positive upstream regulator of the Wnt/β-catenin pathway in gliomagenesis.

## Conclusions

These data suggested that CUX1 was up-regulated in glioma and correlated with poor prognosis. Moreover, CUX1 could behave as an oncogene that stimulated the malignant phenotype (EMT) by activating the Wnt/β-catenin signaling pathway in glioma cells and as a potential target for the development of anti-glioma drugs.

## Data Availability

The original contribution presented in the study are included in the article/Supplementary Material. Future inquiries can be directed to the corresponding author.
