# Supplementary material for: CUX1 Facilitates the Development of Oncogenic Properties Via Activating Wnt/β-Catenin Signaling Pathway in Glioma
Source: Front Mol Biosci. 2021 Aug 6;8:705008. doi: 10.3389/fmolb.2021.705008 (PMC8377541; doi:10.3389/fmolb.2021.705008)
Supplement: Supplementary file 1 [file DataSheet1.docx]

Supplementary Material and Supplementary Figures


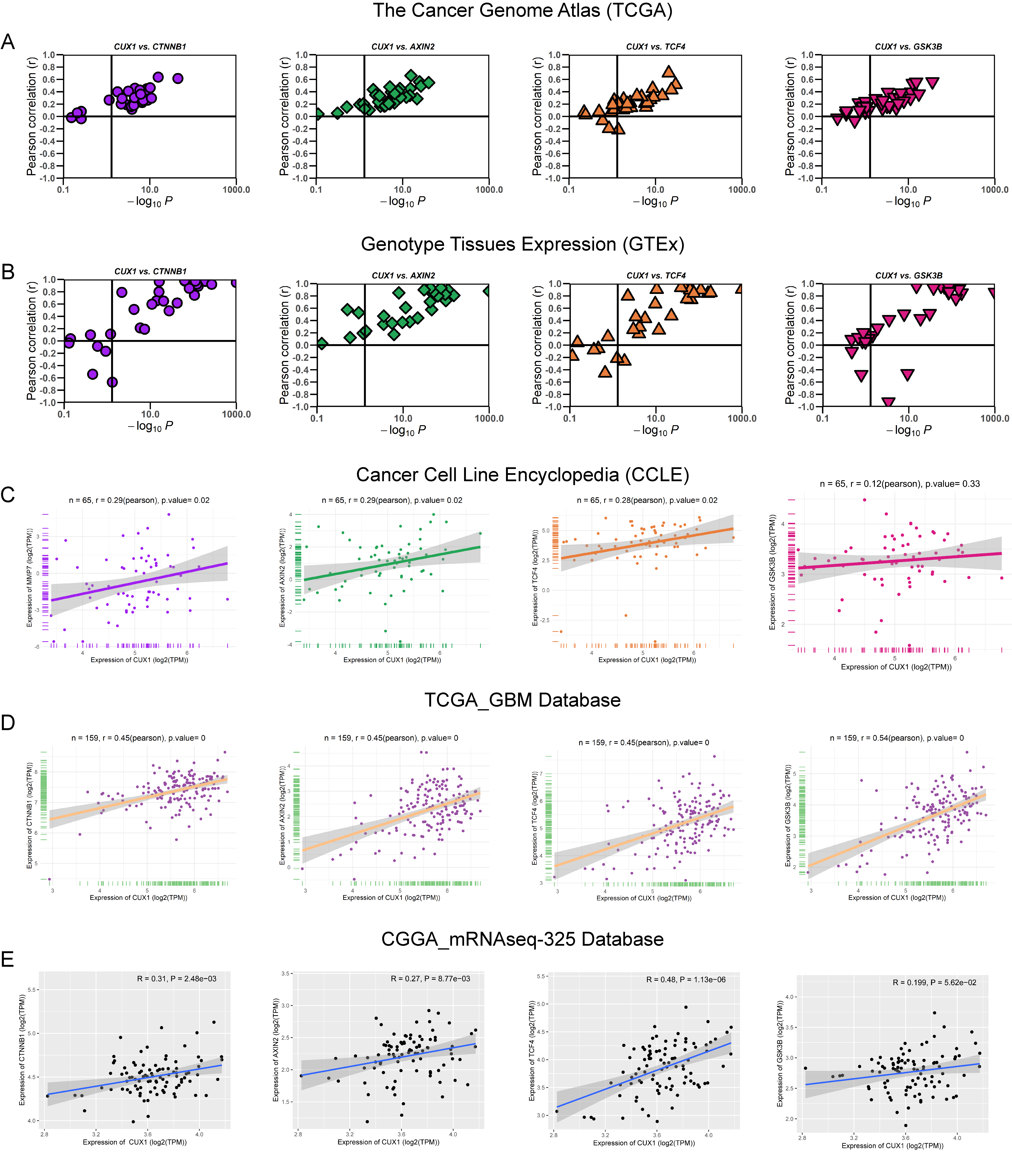


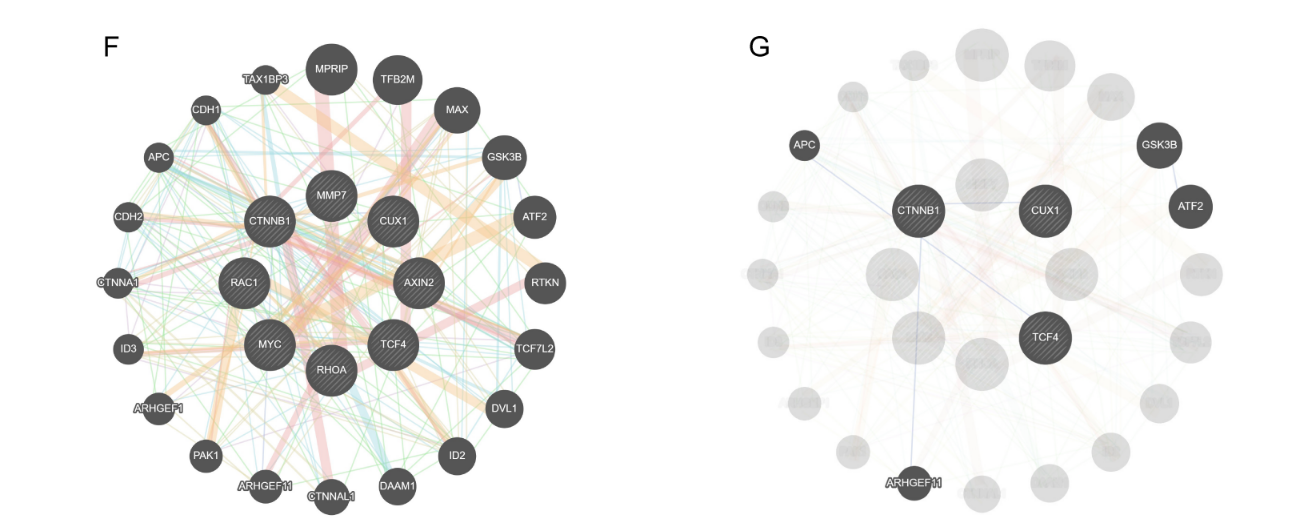


**Supplement Figure**

**Figure 1. Co-expression analysis and interaction network of CUX1. (A-E)** Correlation of CUX1 with several key regulatory factors (CTNNB1, Axin2, TCF4 and GSK3β) of Wnt/β-catenin signaling pathway in normal tissues(A), cancer samples(B), center nerve system cell lines(C) and glioma sample(D-E), based on the data from Genotype Tissue Expression (GTEx), The Cancer Genome Atlas (TCGA), Cancer Cell Line Encyclopedia (CCLE) database and Chinese Glioma Genome Atlas (CGGA) database respectively. **(F-G)** The interaction network of CUX1 protein was analyzed through the GeneMANIA database, CTNNB1, Axin2, GSK3β were found closely related and presented co-expression.

**Supplement Table 1.**

**Expression of CUX1 in normal brain (NB) and different grades gliomas**

| Group | Case | Score of CUX1 expression | | |
| --- | --- | --- | --- | --- |
|  |  | Negative | Low | High |
| NB | 15 | 5（33.3） | 9 (60) | 1 (6.7) |
| Grade Ⅰ-Ⅱ | 30 | 3 (10) | 20 (66.7) | 7 (23.3) |
| Grade Ⅲ-Ⅳ | 50 | 0 (0) | 13 (26) | 37 (74) |

**Supplement Table 2.**

**Correlation between expression of CUX-1 and clinicopathological factors in gliomas**

| Clinicopathological Parameter ,value | | CUX1 expression | | | |
| --- | --- | --- | --- | --- | --- |
|  |  | *N* | Low expression | High expression | *P* value |
| Gender |  |  |  |  |  |
| Male |  | 39(48.8) | 21(53.8) | 18(46.2) |  |
| Female |  | 41(51.2) | 14(34.1) | 27(65.9) | 0.076 |
| Age |  |  |  |  |  |
| <44 |  | 27(33.8) | 11(40.7) | 16(59.3) |  |
| ≥44 |  | 53(66.3) | 24(45.3) | 29(54.7) | 0.699 |
| Tumor diameter | |  |  |  |  |
| <4 cm |  | 16(20.0) | 6(37.5) | 10(62.5) |  |
| ≥4 cm |  | 64(80.0) | 29(45.3) | 35(54.7) | 0.573 |
| Tumor location | |  |  |  |  |
| Frontal |  | 39(48.8) | 19(48.7) | 20(51.3) |  |
| Temporal |  | 19(23.7) | 8(42.1) | 11(57.9) |  |
| Other |  | 22(27.5) | 8(36.4) | 14(63.6) | 0.638 |
| KPS |  |  |  |  |  |
| <80 |  | 42(52.5) | 20(47.6) | 22(52.4) |  |
| ≥80  WHO grade  Ⅰ-Ⅱ  Ⅲ-Ⅳ  MGMT expression  Low  High  P53 expression  Low  High  Ki-67 expression  Low  High | | 38(47.5)  29(36.3)  51(63.7)  48(60.0)  32(40.0)  47(58.8)  33(41.2)  39(48.8)  41(51.2) | 15(39.5)  18(62.1)  17(33.3)  24(50.0)  21(65.6)  27(57.5)  8(24.2)  26(66.7)  9(21.9) | 23(60.5)  11(37.9)  34(66.7)  24(50.0)  11(34.4)  20(42.5)  25(75.8)  13(33.3)  32(78.1) | 0.463  **0.013***  0.168  **0.003***  **0.00006*** |
